# Reward predictions bias attentional selection

**DOI:** 10.3389/fnhum.2013.00262

**Published:** 2013-06-11

**Authors:** Brian A. Anderson, Patryk A. Laurent, Steven Yantis

**Affiliations:** Department of Psychological and Brain Sciences, Johns Hopkins UniversityBaltimore, MD, USA

**Keywords:** selective attention, attentional capture, reward learning, reward prediction, incentive salience

## Abstract

Attention selects stimuli for perceptual and cognitive processing according to an adaptive selection schedule. It has long been known that attention selects stimuli that are task relevant or perceptually salient. Recent evidence has shown that stimuli previously associated with reward persistently capture attention involuntarily, even when they are no longer associated with reward. Here we examine whether the capture of attention by previously reward-associated stimuli is modulated by the processing of current but unrelated rewards. Participants learned to associate two color stimuli with different amounts of reward during a training phase. In a subsequent test phase, these previously rewarded color stimuli were occasionally presented as to-be-ignored distractors while participants performed visual search for each of two differentially rewarded shape-defined targets. The results reveal that attentional capture by formerly rewarded distractors was the largest when both recently received and currently expected reward were the highest in the test phase, even though such rewards were unrelated to the color distractors. Our findings support a model in which value-driven attentional biases acquired through reward learning are maintained via the cognitive mechanisms involved in predicting future rewards.

## Introduction

Perception is limited in its representational capacity, which gives rise to the need to perceive stimuli selectively. Selective attention controls the availability of stimuli for cognition, decision making, and action (Desimone and Duncan, 1995). Recent evidence reviewed below suggests that attentional priority is influenced by prior associations between stimuli and reward, as well as by the current reward value of stimuli. By attending to stimuli associated with the delivery of reward (e.g., nutrients), organisms maximize the opportunity to procure valuable resources that are critical to their survival and wellbeing.

The voluntary deployment of attention can be influenced by the reward value of stimuli. For example, visual search is more efficient for targets associated with the delivery of reward (e.g., Kiss et al., 2009; Kristjansson et al., 2010). Targets associated with high reward are also more robustly represented in early visual areas of the brain (Serences, 2008; Serences and Saproo, 2010).

Certain stimuli capture attention involuntarily, including physically salient stimuli (e.g., Yantis and Jonides, 1984; Theeuwes, 1992, 2010) and stimuli possessing goal-related features (e.g., Folk et al., 1992; Anderson and Folk, 2012). Recent evidence demonstrates that attention is also captured by previously rewarding stimuli. The recent delivery of high reward primes attention to a reward-associated stimulus (Hickey et al., 2010, 2011). Furthermore, we have shown that stimuli persistently capture attention after repeated pairings with reward, even when they are no longer rewarded and are otherwise inconspicuous and task-irrelevant (Anderson et al., 2011b; Anderson and Yantis, 2012, 2013).

Over the past two decades, much has been learned about the underlying neurobiology of reward. Learned reward predictions are represented in the basal ganglia (BG), such that the onset of reward-associated stimuli elicits the release of dopamine (DA) from BG neurons (Schultz et al., 1997; Waelti et al., 2001). It is also known that unexpected reward also elicits phasic DA release. Once an organism learns that a stimulus predicts reward, the receipt of the expected reward no longer produces DA release in response to the reward; instead, the omission of the expected reward depresses DA activity (Schultz et al., 1997). Thus, phasic DA activity is thought to convey a signal that represents both reward prediction and reward-prediction error.

The relationship between the underlying mechanisms for processing reward and for biasing attention in favor of reward-associated stimuli is unknown. One possibility is that reward motivates the recruitment of different cognitive processes, such as memory storage and perceptual learning, in order to establish and maintain attentional biases that prove adaptive in promoting reward procurement. By such an account, value-driven attentional biases are maintained independently of the cognitive architecture that subserves reward processing. Another possibility is that value-driven attentional priority is represented and signaled by the reward processing system, which is sensitive to current reward predictions. In the present study, we adjudicate between these two accounts by measuring the magnitude of attentional capture by previously reward-associated stimuli when different levels of reward were predicted from the current task.

**Figure 1:**
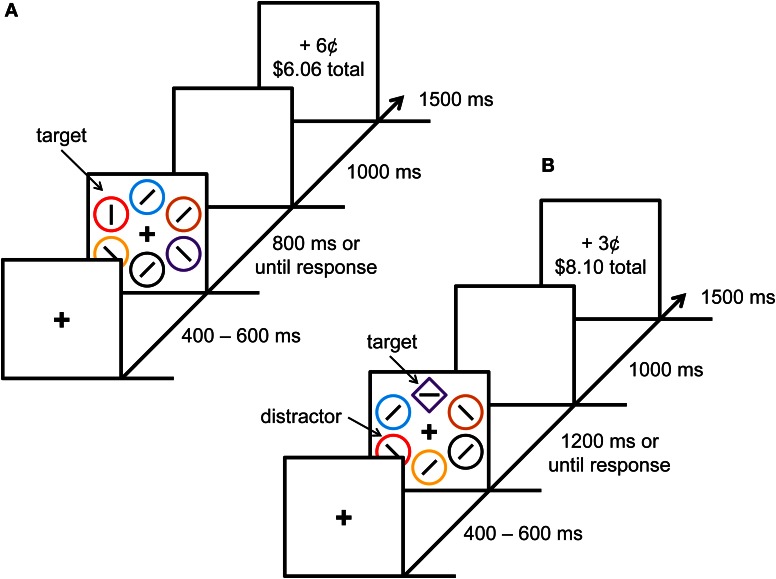
**Experimental paradigm**. Sequence of events and time course for a trial during the training phase **(A)** and test phase **(B)**. Each trial was followed by a blank 1000 ms intertrial interval.

The experiment was modeled after the experiments of Anderson et al. (2011b) and included a training phase and a test phase. In the training phase, participants learned to associate each of two color stimuli with different amounts of monetary reward (see Figure 1A). The training phase was followed by a test phase that was a modified version of the additional singleton paradigm (Theeuwes, 1992) in which the target of visual search was a shape singleton (see Figure 1B). Reward feedback was also provided in the test phase, and one of the shape targets (e.g., a unique circle among diamonds) was probabilistically associated with more reward than the other. This reward structure allowed participants to experience reward prediction and reward-prediction error on each trial. Each item in the test phase was rendered in a different color, but color was not relevant to the task. However, one of the non-targets was sometimes rendered in a color that was associated with reward during the preceding training phase. This design allowed us to assess the magnitude of value-driven attentional capture by previously rewarded colors in the test phase, as a function of both reward prediction and reward-prediction error. The hypothesis that value-driven attentional priority is represented and signaled by the reward processing system predicts that value-driven attentional capture should be maximal when these reward signals are the largest, even though current rewards are unrelated to the previously reward-associated stimuli.

## Materials and methods

### Participants

Sixteen participants were recruited from the Johns Hopkins University community. All were screened for normal or corrected-to-normal visual acuity and color vision. Participants were provided monetary compensation based on performance that varied between 12 and $15 (mean = $13.44). All procedures were approved by the Johns Hopkins University Institutional Review Board and all participants provided informed consent.

### Apparatus

A Mac Mini equipped with Matlab software and Psychophysics Toolbox extensions was used to present the stimuli on a Dell P991 monitor. The participants viewed the monitor from a distance of approximately 50 cm in a dimly lit room. Manual responses were entered using a 101-key US layout keyboard.

### Stimuli

Each trial consisted of a fixation display, a search array, and a feedback display (see Figure 1). The fixation display contained a white fixation cross (0.5 × 0.5° visual angle) presented in the center of the screen against a black background, and the search array consisted of the fixation cross surrounded by six shape stimuli (each with a diameter of 2.3° visual angle) placed at equal intervals on an imaginary circle with a radius of 5°. The six shapes were each rendered in a different color (red, green, blue, cyan, pink, orange, yellow, or white).

During the training phase, all six of the shapes were circles and the target was defined as the red or green circle (exactly one of which was presented on each trial). During the test phase, the six shapes consisted of either a diamond among circles or a circle among diamonds, and the target was defined as the unique shape. On 25% of the trials in the test phase, one of the non-target shapes was red and on another 25% of the trials, one of the non-target shapes was green; these constituted the formerly rewarded distractors (these two non-target shapes are referred to as “distractors”). The target was never red or green during the test phase.

Inside the target shape, a white line segment was oriented either vertically or horizontally, and inside each of the non-targets, a white line segment was tilted at 45° to the left or to the right (randomly for each non-target). The participant was required to report whether the orientation of the line segment inside the target shape was vertical or horizontal with a corresponding key press. Correct responses were followed in both phases of the experiment by a feedback display that informed participants of the monetary reward earned on that trial, as well as the total reward accumulated thus far in the experiment.

### Design and procedure

The experiment consisted of 240 trials during each of the two phases, for a total of 480 trials. Participants completed 50 practice trials prior to the training phase, and 20 practice (distractor-absent) trials prior to the test phase; behavioral data from these practice trials were not included in any analysis. In the training phase, target identity and target location were fully crossed and counterbalanced, and trials were presented in a random order. In the test phase, target identity, target location, distractor identity, and distractor location were fully crossed and counterbalanced, and trials were presented in a random order. Thus, in the test phase, the presence and identity of the distractor provided no predictive information concerning the target or reward.

In both the training and test phase, one of the two targets (e.g., red during training and diamond singleton at test) was followed by a high reward on 80% of correct trials and a low reward on the remaining 20%; the percentages were reversed for the low-reward target. High and low rewards were 6 and 2¢, respectively, during the training phase and 3 and 1¢ during the test phase (higher rewards were used in the training phase to maximize the learning of the color–reward associations). Incorrect responses or responses that were too slow were followed by feedback indicating 0¢ had been earned. Which color target and shape-singleton target was associated with high reward in their respective phase of the experiment was counterbalanced across participants, such that each combination of color and shape was used equally often. Participants were not informed of the reward contingencies, which had to be learned through experience in the task. Upon completion of the experiment, participants were given the cumulative reward they had earned.

Each trial began with the presentation of the fixation display for a randomly varying interval of 400, 500, or 600 ms. The search array then appeared and remained on screen until a response was made or the trial timed out. Trials timed out after 800 ms in the training phase and 1200 ms in the test phase. The search array was followed by a blank screen for 1000 ms, the reward feedback display for 1500 ms, and a 1000 ms intertrial interval.

Participants made a forced-choice target identification by pressing the “z” and the “m” keys for the vertically- and horizontally-orientated targets, respectively. If the trial timed out, the computer emitted a 500 ms and 1000 Hz feedback tone.

### Data analysis

Only correct responses were included in all analyses of RT, and all RTs more than three standard deviations above or below the mean of their respective condition for each participant were excluded.

## Results

### Training phase

There were no significant differences in RT [*t*_(15)_ = −0.16, *p* = 0.877] or accuracy [*t*_(15)_ = −1.04, *p* = 0.316] to report a high-reward target compared to a low-reward target (means for high-reward target: 537 ms, 90.0%; means for low-reward target: 536 ms, 91.1%). There were also no significant differences in RT [*t*_(15)_ = 1.81, *p* = 0.091] or accuracy [*t*_(15)_ = −1.14, *p* = 0.272] based on the color of the target (means for red: 534 ms, 91.2%; means for green: 539 ms, 89.9%). In our prior studies on reward and attention, participants have generally been faster to respond to high-reward targets than to low-reward targets (Anderson et al., 2011a, 2012; Anderson and Yantis, 2012). The present results suggest that top–down attentional control dominated performance in the training phase, such that participants searched for the two target colors with approximately equal priority. The reward feedback allowed participants to learn the color–reward contingencies, however, and the effects of these contingencies on performance in the test phase were of primary interest.

### Test phase

We first compared RT and accuracy for trials containing a high-reward target compared to a low-reward target, as in the training phase. As in the training phase, RT [*t*_(15)_ = −0.28, *p* = 0.785] and accuracy [*t*_(15)_ = −0.26, *p* = 0.798] did not differ based on the value of the target (means for high-reward target: 673 ms, 89.8%; means for low-reward target: 663 ms, 90.6%). There was a highly significant effect of target shape, such that participants were substantially faster and more accurate to report circle-singleton targets compared to diamond-singleton targets [for RT: mean difference = 130 ms, *t*_(15)_ = 14.26, *p* < 0.001, *d* = 3.57; for accuracy: mean difference = 8.9%, *t*_(15)_ = 4.59, *p* < 0.001, *d* = 1.15]. This suggests that the circle singleton was more physically salient than the diamond singleton.

Next, to assess the effect of distractor presence, trials during the test phase were sorted according to whether they contained a non-target formerly associated with high reward (high-value distractor, 25% of trials), a non-target formerly associated with low reward (low-value distractor, 25% of trials), or neither (50% of trials). A repeated measures ANOVA revealed that RT in the three distractor conditions differed significantly [Table 1, *F*_(2, 30)_ = 16.63, *p* < 0.001, η^2^_*p*_ = 0.526]. Neither the color that was associated with high reward during training [*F*_(2, 24)_ = 2.93, *p* = 0.073] nor the shape singleton that was associated with high reward at test (*F* < 1) interacted significantly with the effect of distractor condition on RT, and the three-way interaction was also not significant [*F*_(2, 24)_ = 1.08, *p* = 0.357], so we collapsed across these two factors. A *post-hoc* contrast revealed that RT was slower when a previously rewarded color distractor was present compared to the distractor-absent condition, indicating the presence of value-driven attentional capture by formerly rewarded but now irrelevant colors [*t*_(15)_ = 5.72, *p* < 0.001, *d* = 1.72]; RT did not differ between the high- and low-value distractor conditions [*t*_(15)_ = −0.63, *p* = 0.537], and the distractors captured attention regardless of their color [both *t*'s > 4.50, *p*'s < 0.001]. Accuracy did not differ significantly among the three conditions (*F* < 1), nor did the effect of distractor condition on accuracy interact with the color associated with high-reward during training [*F*_(2, 24)_ = 2.03, *p* = 0.153] or the shape singleton associated with high-reward at test (*F* < 1), and the three-way interaction was also not significant (*F* < 1).

**Table 1:** **Response time and accuracy by condition in the test phase**.

	**Distractor condition**		
	**Absent**	**Low-value**	**High-value**
Response time (ms)	652	677	674
Accuracy	89.4%	91.1%	90.0%

**Figure 2:**
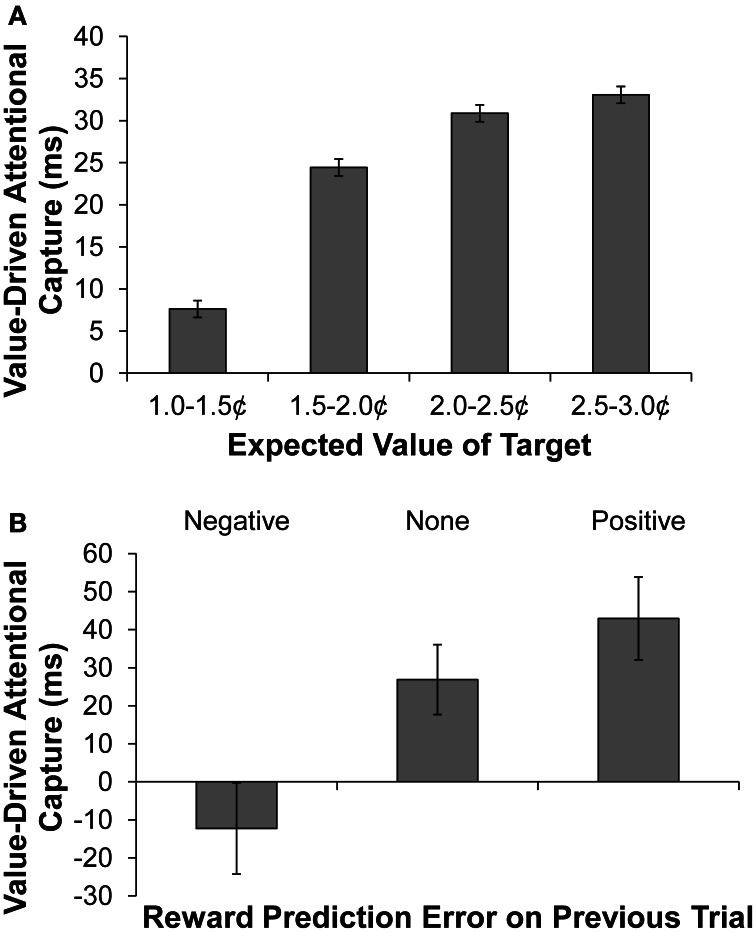
**Behavioral results. (A)** Value-driven attentional capture (defined as the mean difference in RT between distractor-present and distractor-absent trials) as a function of the value of the search context (defined as the mean reward obtained on the previous 5 trials in which the current shape singleton served as the target). **(B)** Value-driven attentional capture as a function of the reward-prediction error experienced on the previous trial. The error bars reflect the within-subjects SEM.

According to reward-prediction error accounts, a representation of current expected reward develops based on a trial's former context (e.g., Nakahara et al., 2004). We therefore next examined how the magnitude of predicted reward on a given trial (based on the target's shape) modulated the degree to which the formerly rewarded color distractors captured attention. The predicted reward on a given trial was defined as the mean reward received over the previous five trials in which the current shape-singleton target served as the target. This computed value was used rather than the actual reward probabilities assigned to the singleton target, as previous research has shown that participants are highly sensitive to recent reward history (e.g., Serences, 2008), and this method better accounts for trials in the early part of the test phase in which participants have had little experience with the current reward contingencies. The mean reward received in the last 5 trials is, of course, highly correlated with the actual reward probabilities. But estimated value can vary considerably given the stochastic fluctuations in actual reward delivery, and so this method provides a potentially more sensitive index of the influence of experienced value on performance. We calculated value-driven attentional capture (slowing of RT on distractor present vs. absent trials) separately for trials on which the current shape singleton's predicted reward fell into one of four equally-spaced ranges as shown in Figure 2A. The magnitude of value-driven capture differed significantly for different amounts of predicted reward [*F*_(3, 45)_ = 2.96, *p* = 0.042, η^2^_*p*_ = 0.165], and the data were well accounted for by a linear trend in which the magnitude of capture becomes greater as predicted reward increases [*F*_(1, 15)_ = 6.97, *p* = 0.019, η^2^_*p*_ = 0.317].

RT on distractor-absent trials did not differ based on predicted reward (*F* < 1), meaning that the observed changes in the magnitude of value-driven attentional capture as a function of predicted reward were not the consequence of baseline shifts in RT (the mean RTs on distractor-absent trials as a function of increasingly high predicted reward were 652, 646, 640, and 664 ms). Neither did the magnitude of value-driven attentional capture differ based on the mean reward received over the previous 5 trials without respect to the current target (*F* < 1), meaning that the effect of predicted reward on attentional capture did not reflect a more global consequence of recently received rewards.

In addition to the mean reward received over the last few trials a given stimulus was a target, another potentially salient reward-related signal concerns recent reward-prediction error. Positive reward-prediction error occurs when more reward is received than predicted, and negative reward-prediction error when less reward is received than expected. Reward-prediction errors are thought to provide a teaching signal that adjusts subsequent reward predictions to reduce the discrepancy between previously predicted and received reward (e.g., Waelti et al., 2001). Therefore, the representation of reward on a given trial can be expressed in terms of the reward-prediction error realized on the preceding trial, with the magnitude being larger following positive reward-prediction error and smaller following negative reward-prediction error.

A positive reward-prediction error was taken to occur when participants received a high reward following a singleton target that typically yields low reward, and a negative reward-prediction error was taken to occur when participants received a low reward following a singleton target that usually yields high reward. We found that the magnitude of value-driven attentional capture differed significantly following the three possible outcomes of reward prediction on the previous trial (positive prediction error, negative prediction error, and no prediction error) [Figure 2B, *F*_(2, 30)_ = 4.63, *p* = 0.018, η^2^_*p*_ = 0.236]. In particular, value-driven capture was significantly greater following a positive reward-prediction error than following a negative reward-prediction error [*t*_(15)_ = 2.63, *p* = 0.019, *d* = 0.66]; the former produced substantial value-driven attentional capture, while the latter produced no evidence of attentional capture. RT on distractor-absent trials did not differ based on the reward-prediction error on the preceding trial (*F* < 1), meaning that the observed changes in the magnitude of value-driven attentional capture were not the consequence of baseline shifts in RT (the mean RTs on distractor-absent trials following negative, no, and positive reward-prediction error were 658, 653, and 641 ms, respectively). This provides further evidence that the attentional bias toward stimuli with learned value varies as a function of ongoing task-related reward processing.

## Discussion

Attention selects stimuli for perceptual and cognitive processing. By attending to stimuli associated with the delivery of reward, organisms maximize the opportunity to procure valuable resources. We have previously shown that valuable stimuli capture attention involuntarily (Anderson et al., 2011a,b, 2012; Anderson and Yantis, 2012, 2013). The present study tested the hypothesis that this attentional bias for valuable stimuli is maintained via the cognitive mechanisms involved in processing rewards.

Using two different measures of ongoing reward processing, we found strong influences of both currently expected reward and recent reward-prediction error on the magnitude of value-driven attentional capture by formerly rewarded distractors. The greater the reward prediction on a given trial, the greater the distraction caused by previously rewarded but currently irrelevant stimuli. This finding is surprising because one might hypothesize participants to be most resistant to distraction when a high reward target was available to motivate goal-directed performance. Value-driven attentional capture was also more pronounced following positive reward-prediction error (i.e., when more reward was received than expected) than following negative reward-prediction error. This finding is also somewhat surprising because one might hypothesize that the reward-prediction errors would increase attention to the target, rather than to the distractor. Instead, this result shows that when an unexpectedly high reward has been obtained, stimuli that predict high reward in both the current and past contexts tend to capture attention.

Interestingly, value-driven attentional capture was small or non-existent when predicted reward was low and recent reward-prediction error was negative, respectively. This contrasts with the magnitude of value-driven attentional capture typically observed without reward feedback during the test phase (e.g., Anderson et al., 2011a,b; Anderson and Yantis, 2012, 2013). This suggests that relatively small rewards are experienced as disappointing and result in a reduction in the attentional bias afforded to reward-associated stimuli, consistent with the small or even negative priming observed following a low reward (e.g., Della Libera and Chelazzi, 2006; Hickey et al., 2010, 2011).

These behavioral results suggest the existence of a common mechanism that represents both reward predictions and reward-prediction error, and signals incentive salience (i.e., attentional priority for formerly rewarded stimuli). One candidate for this mechanism is the phasic DA signal in the BG (Schultz et al., 1997; Waelti et al., 2001). This is consistent with recent evidence showing that the visual representation of a reward-associated cue is modulated by the receipt of unrelated reward and corresponding reward-related DA activity (Arsenault et al., 2013). If value-based attentional priority is signaled via mechanisms that overlap with the signaling of current reward, modulating the representation of current reward should produce concurrent modulations in value-driven attentional capture. By relating ongoing reward processing to value-driven attentional capture in this way, our findings provide further insight into the mechanisms underlying attentional capture by reward-associated stimuli, which, in turn, has important implications for theories linking reward learning to attentional control (e.g., Della Libera and Chelazzi, 2006, 2009; Serences, 2008; Peck et al., 2009; Raymond and O'Brien, 2009; Hickey et al., 2010, 2011; Krebs et al., 2010; Serences and Saproo, 2010; Anderson et al., 2011a,b, 2012; Della Libera et al., 2011; Anderson and Yantis, 2012, 2013; Hickey and van Zoest, 2012).

It is worth noting that in the present study, the magnitude of attentional capture by stimuli previously associated with reward did not depend on the magnitude of prior reward value experienced during training (i.e., RT did not differ between the high- and low-value distractor conditions). One possibility is that the reward associated with the color distractors was influenced by the reward received in the test phase, which was unrelated to color. Both color targets were associated with reward outcome in the training phase of present study, and the extent to which persistent reward-related attentional biases acquired through learning should scale with the magnitude of prior reward is unclear. Previous studies show that reward associations play a direct and important role in the development of attentional biases for former targets (Anderson et al., 2011a,b, 2012; Anderson and Yantis, 2013), which, together with the observed influence of ongoing rewards, suggests that the observed attentional biases for former targets reflects an effect of reward history.

Our findings also provide further evidence for a mode of attentional control that is distinct from the well-documented stimulus-driven and goal-directed mechanisms (e.g., Folk et al., 1992; Theeuwes, 1992, 2010; Yantis, 2000; Connor et al., 2004). We show that previously reward-predictive but currently irrelevant stimuli capture attention even when they are not task relevant and not physically salient, replicating previous results (Anderson et al., 2011b; Anderson and Yantis, 2012, 2013). Our data also reveal that motivating current task goals with reward potentiates rather than minimizes attentional capture by previously valuable but currently irrelevant stimuli. If value-driven attentional capture merely reflected difficulty overcoming a previously motivated selection strategy, it would not be expected to be modulated in this way and might instead be better overcome by the motivation provided by currently expected reward. Thus, our results provide direct evidence that learned value influences attentional control in a way that does not depend on either physical salience or ongoing goals, and is instead mediated by the cognitive mechanisms involved in reward processing.

Attentional biasing of reward-associated stimuli is adaptive in many circumstances, facilitating the procurement of future rewards. However, previous reward learning and ongoing goals will at times conflict, as they do, for example, in the case of desired abstinence from a substance of abuse. Visual cues for a substance of abuse can involuntary capture attention in drug-dependent populations (e.g., Lubman et al., 2000; Marissen et al., 2006; Field and Cox, 2008), much as the previously reward-associated distractors capture attention in the present study. This drug-related attentional bias is thought to play an important role in contributing to relapse following periods of abstinence (see Field and Cox, 2008, for a review). Our findings have implications for theories of such disordered attentional control in addiction by demonstrating that reward-related attentional biases are mediated specifically by the brain mechanisms involved in processing rewards, which are known to be directly affected by drugs of abuse (e.g., Berridge and Robinson, 1998).

### Conflict of interest statement

The authors declare that the research was conducted in the absence of any commercial or financial relationships that could be construed as a potential conflict of interest.
